# Feasibility and Efficacy of the Addition of Heart Rate Variability Biofeedback to a Remote Digital Health Intervention for Depression

**DOI:** 10.1007/s10484-020-09458-z

**Published:** 2020-04-03

**Authors:** Marcos Economides, Paul Lehrer, Kristian Ranta, Albert Nazander, Outi Hilgert, Anu Raevuori, Richard Gevirtz, Inna Khazan, Valerie L. Forman-Hoffman

**Affiliations:** 1 Meru Health Inc, Palo Alto, CA USA; 2 Rutgers Robert Wood Johnson Medical School, Piscataway, NJ USA; 3 Department of Adolescent Psychiatry, Helsinki University Central Hospital, Helsinki, Finland; 4 Clinicum, Department of Public Health, University of Helsinki, Helsinki, Finland; 5 Department of Clinical Psychology, California School of Professional Psychology, Alliant University, San Diego, USA; 6 Harvard Medical School, Boston Center for Health Psychology and Biofeedback, Boston, USA

**Keywords:** Depression, Heart rate variability, Biofeedback, Smartphone app, Online intervention, Digital health, mHealth, Mindfulness, Meditation

## Abstract

A rise in the prevalence of depression underscores the need for accessible and effective interventions. The objectives of this study were to determine if the addition of a treatment component showing promise in treating depression, heart rate variability-biofeedback (HRV-B), to our original smartphone-based, 8-week digital intervention was feasible and whether patients in the HRV-B (“enhanced”) intervention were more likely to experience clinically significant improvements in depressive symptoms than patients in our original (“standard”) intervention. We used a quasi-experimental, non-equivalent (matched) groups design to compare changes in symptoms of depression in the enhanced group (n = 48) to historical outcome data from the standard group (n = 48). Patients in the enhanced group completed a total average of 3.86 h of HRV-B practice across 25.8 sessions, and were more likely to report a clinically significant improvement in depressive symptom score post-intervention than participants in the standard group, even after adjusting for differences in demographics and engagement between groups (adjusted OR 3.44, 95% CI [1.28–9.26], *P* = .015). Our findings suggest that adding HRV-B to an app-based, smartphone-delivered, remote intervention for depression is feasible and may enhance treatment outcomes.

**Electronic supplementary material:**

The online version of this article (10.1007/s10484-020-09458-z) contains supplementary material, which is available to authorized users.

The global burden of depression, the world’s leading cause of disability (Geneva: World Health Organization 2017), is undeniably large. Increased risk of other comorbid mental and physical disorders, decreased functioning, quality of life and productivity, and increased risk of early mortality drive the yearly $210.5 billion economic burden of depression in the U.S. alone (Greenberg et al. 2015). Despite the availability of several evidence-based treatments, the prevalence of depression has continued to rise—an estimated 18% between 2005 and 2015 (Kessler 2012; McCall and Kintziger 2013; National Council for Behavioral Health 2017). The chronic and recurrent nature of depression further underscores the critical need for effective, long-lasting interventions that those affected can easily access.

Current treatments for depression typically include either psychotherapy and/or medication (Huhn et al. 2014), which require highly trained professionals to administer. When treated with antidepressants, an estimated 30–50% of patients do not show significant symptom improvement (El-Hage et al. 2013), up to 80% report at least one side effect (Wang et al. 2018), and at least 50% of patients are likely to relapse within 6- to 12-months of treatment withdrawal (Baldessarini et al. 2015; Johansson et al. 2015). Shortages in trained providers have brought even more attention to the urgent need to provide alternative solutions (National Council for Behavioral Health 2017). Self-directed therapies that can be easily learned, mastered, and utilized by depressed patients might help address some of these access issues. Internet- and smartphone-based depression treatments delivered with minimal guidance have demonstrated clinical effectiveness (Firth et al. 2017; Richards and Richardson 2012), with effect sizes similar to face-to-face interventions (Carlbring et al. 2018).

As such, we created the *Meru Health* online digital clinic to deliver a comprehensive 8-week intervention to patients via a Smartphone app. The program is overseen by a remote licensed therapist, who monitors progress via data collected in the app and provides feedback as needed. Content delivered in the weekly modules is derived from several evidence-based treatments for depression including Mindfulness-Based Meditation (MM) (Kabat-Zinn 1994; Morgan 2003), Cognitive-Behavioral Therapy (CBT) (Beck 1979), and Behavioral Activation Therapy (BAT) (Jacobson et al. 2001) delivered via texts, videos, and daily practices. Analyses of this program have indicated post-intervention clinically significant reductions in depressive symptoms that remain at 6- and 12-month follow-up assessments (Economides et al. 2019; Goldin et al. 2019).

As part of making continuous improvements to the *Meru Health* intervention, we recently added another self-directed component to the program—heart rate variability biofeedback (HRV-B). Heart rate variability (HRV) describes the variation in interbeat time intervals among heart beats, and is believed to be a marker of the body’s ability to self-regulate during times of distress via interactions between the cardiac and emotional control centers of the body (Sztajzel 2004). Low levels of HRV have been found for a number of medical conditions, as well as cognitive and mental health problems (Camm et al. 1996; Friedman 2007; Thayer and Lane 2009; Thayer and Sternberg 2006), including depression (Kemp et al. 2010). As such, therapies that increase HRV strengthen various modulatory reflexes in the cardiovascular system and improve emotional regulation, thus providing benefit to those suffering from these conditions (Lehrer and Eddie 2013). One such therapy is HRV-B, which involves learning a breathing technique that maximizes HRV while monitoring progress throughout the training (Gevirtz 2013; Lehrer and Gevirtz 2014). Potential mechanisms of therapeutic effect involve HRV-B strengthening the parasympathetic system, the baroreflex (one of the body’s homeostatic mechanisms that helps to maintain a relatively constant blood pressure), and/or the inflammation response, such that the body is better able to respond to, regulate, and recover from a distressing event (Lehrer and Gevirtz 2014). Regular practice of this technique has thus been proposed to help mitigate the stress response when faced with a stressful situation (Yu et al. 2018).

Several preliminary studies have reported a therapeutic benefit of HRV-B for patients with high levels of anxiety, stress, and depression (Blasé et al. 2016; Gevirtz 2013; May et al. 2019; Siepmann et al. 2014; van der Zwan et al. 2019). Thus, clinicians have begun integrating HRV-B into existing depressive treatments (Gevirtz 2015). One small trial showed that adding HRV-B to psychotherapy improved post-intervention depressive symptom outcomes significantly more than psychotherapy alone (Caldwell and Steffen 2018). Based on these promising findings and with the understanding that, to our knowledge, there has not been an investigation of whether HRV-B can be integrated into a depression intervention delivered remotely, we conceived the present analysis of our real-world data. Our main objectives were to determine (1) if the addition of HRV-B to our mobile health depression intervention delivered via a Smartphone app (“enhanced program”) is feasible, and (2) whether patients in our enhanced program were more likely to experience clinically significant improvement in depressive symptoms compared with patients in our original (“standard”) program with no HRV-B component, after controlling for differences in engagement. We hypothesized that the enhanced program patients would be able to complete the HRV-B practices and engage in our remote program and, compared to patients enrolled in the standard program, be more likely to have clinically significant improvements in depressive symptoms. As a secondary aim, we evaluated whether the degree of intervention engagement, and specifically HRV-B practice, predicted symptom change.

## Material and Methods

### Study Design

We used a quasi-experimental, pre- and post-intervention, non-equivalent groups design to compare an HRV-B enhanced version of the *Meru Health* intervention (“enhanced”) to historical outcome data from the standard *Meru Health* intervention (“standard”). For each group, symptoms of depression were measured pre-intervention (“baseline”), during weeks 1, 3, 5 and 7 of the intervention, and at the end of the 8-week intervention (“post-intervention”).

### Participants

The present study included adult patients treated at the *Meru Health* online clinic, a national remote healthcare provider that currently operates in the US and Finland. The enhanced group consisted of 48 participants that took part in the HRV-B enhanced intervention between October 2018 and March 2019. Each participant in the enhanced group was matched to a single participant that had previously engaged with the standard *Meru Health* intervention (which did not include HRV-B). Standard group participants were selected from a pool of 76 participants that took part in the *Meru Health* intervention between April 2017 and January 2019. We used 1:1 nearest-neighbor matching (using the *knnsearch* function in Matlab® R2016b) without replacement and used baseline PHQ-9 score and antidepressant status as covariates, as we conjectured these would have the largest impact on depression symptoms over time. This resulted in 48 participants in the standard group that took part in the intervention between October 2017 and January 2019 selected for analysis.

Participants were recruited via online Facebook advertisements that sought individuals for a smartphone-based intervention (SBI) for depression that included self-guided smartphone-delivered content, private access to a therapist via messaging, and an anonymous group chat feature amongst participants. Importantly, the method of recruitment and messaging of the advertisements was identical for both groups. Participants were given free access to the app and trained on how to use the anonymous group chat feature, as well as how to communicate with their assigned therapist, prior to beginning the intervention. Participant demographics (age, gender, and antidepressant status) were acquired prior to the intervention via an intake questionnaire administered online. Outcome measures were administered via the *Meru Health* smartphone app, except for baseline measures which were acquired during screening. Since there is a variable delay between screening and the start of the intervention, baseline scores are completed up to a maximum of 30 days prior to the intervention start date. Post-intervention scores were considered valid if reported during or within 2 weeks following the intervention end-date.

For inclusion, participants had to provide informed consent via the *Meru Health* app, own a smartphone, have at least moderate symptoms of depression (a score ≥ 10 on the Patient Health Questionnaire [PHQ-9] at baseline), and acknowledge/demonstrate the ability to commit to a minimum of 20 min of practice per day, for 6 days per week, across the 8-week intervention (as judged by both the participant and their assigned therapist). Exclusion criteria included a previous suicide attempt, severe active suicidal ideation with a specific plan, severe self-harm, active substance abuse, or a history of psychosis. Inclusion/exclusion criteria were assessed prior to enrolment via phone-based screening interviews between study participants and intervention therapists, as per the standard treatment procedure at the online clinic. Participants were not compensated for their time but could participate in the intervention for free. Data were collected as part of the standard *Meru Health* intervention, and all procedures used were reviewed by Pearl IRB, who granted institutional review board exemption for analyses of previously collected and de-identified data, deemed as having been performed in accordance with the 1964 Helsinki declaration and its later amendments or comparable ethical standards. All participants provided informed consent for their anonymized data to be used for research purposes prior to participation.

### Intervention

#### Standard Intervention

The standard *Meru Health* intervention has been described in detail previously (Economides et al. 2019; Goldin et al. 2019). Briefly, the intervention consists of 8 modules delivered sequentially over an 8-week period, that include content derived from evidence-based practices such as Mindfulness-Based Stress Reduction (MBSR) (Kabat-Zinn 1994), Mindfulness-Based Cognitive Therapy (MBCT) (Morgan 2003), Cognitive-Behavioral Therapy (CBT) (Beck 1979), and Behavioral Activation Therapy (BAT) (Jacobson et al. 2001). The content includes text, video, audio-guided mindfulness meditation (MM) exercises, infographics that illustrate CBT principles, and journal prompts. Daily content and practices range from 10 to 30 min, except for the first day of each week, in which a series of introductory videos extend the content to a maximum of 45 min. The intervention also includes anonymous peer support via a group discussion board, and support by a licensed Meru Health-employed remote therapist. Therapists review participant engagement and self-reported outcomes throughout the intervention and provide 1:1 support to participants via messaging (and less frequently, phone calls). Participants can also message therapists directly when needed.

#### Enhanced Intervention

**Fig. 1:**
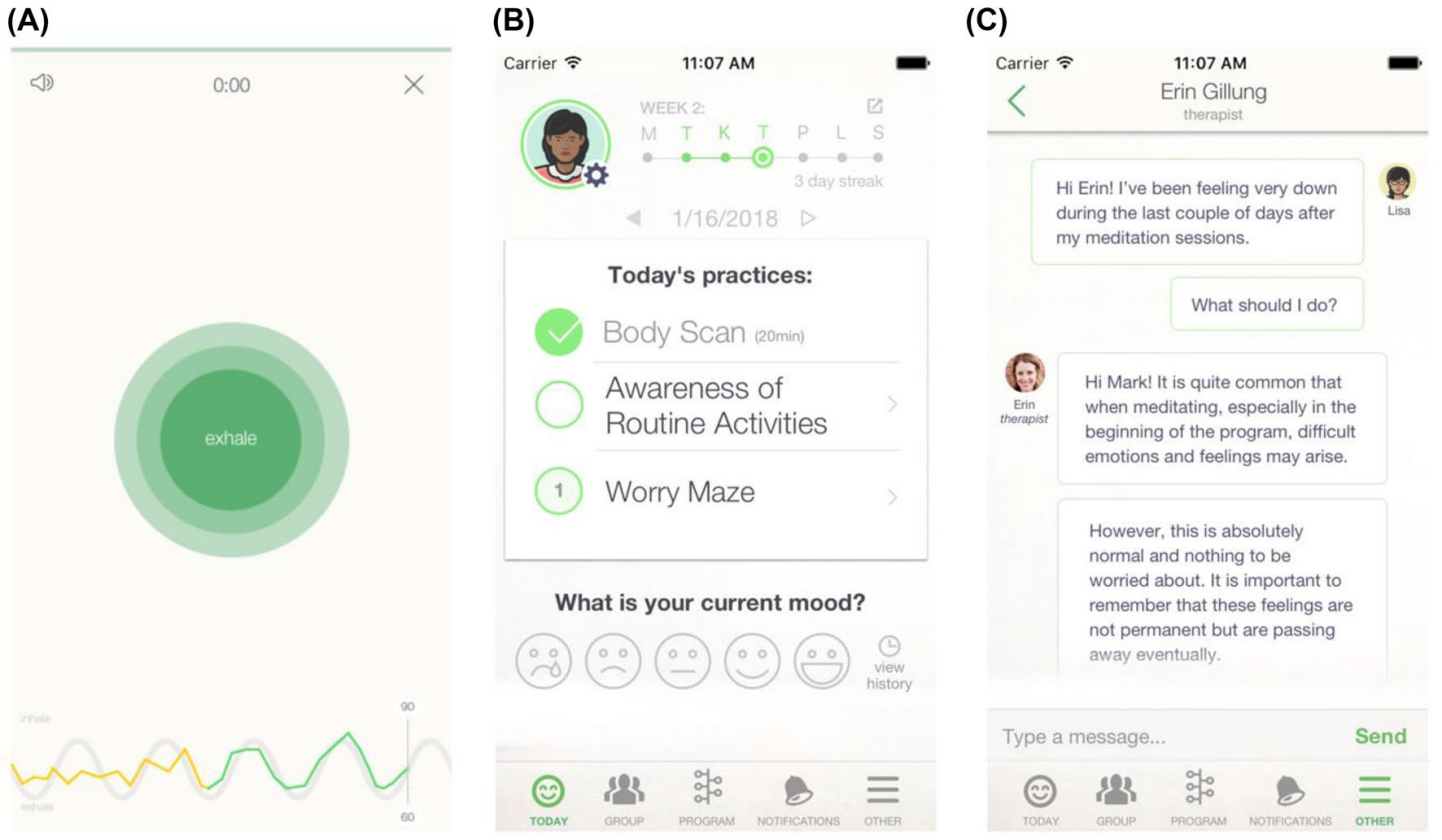
Screenshots of the Meru Health application. The screenshots depict HRV-B practice in the enhanced group (**a**), daily practices common to both groups (**b**), and messaging between patient and therapist (**c**). The colored heart rate tracer in the HRV-B practice (bottom of panel **a**) displays amber during period of low resonance and green during periods of high resonance

The enhanced intervention consists of the same core modules, structure and content as the standard intervention, with the addition of daily HRV-B exercises, referred to as “resonance breathing” within the Meru Health app (see Fig. 1). These exercises start at a duration of 5 min and gradually increase up to a maximum of 20 min from week 4 of the intervention onwards (though participants could adjust the duration in 5-min increments according to their preference). To accommodate the HRV-B exercises, the enhanced intervention features 2 h and 56 min less MM content than the standard intervention (though in practice this difference can vary according to the number of days in which participants engage with the intervention, and whether any MM sessions are completed more than once).

Participants self-administered HRV-B via the Meru Health app using a HeartMath® Bluetooth photoplethysmography (PPG) sensor, which was sent to each participant prior to starting the intervention. The sensor collected data either from an earlobe or from a fingertip. Each participant received a brief written introduction to HRV and resonance breathing, including how to use the sensor, and was required to complete introductory content via the *Meru Health* app, during which they could set a daily time / reminder to engage with the practice. During each HRV-B session, participants were guided by a visual pacer that expands during inhalation for 4 s and contracts during exhalation for 6 s, achieving resonance breathing at rate of approximately 6 breaths per minute. The visual pacer was supplemented with recorded breaths that matched the rate of the visual pacer. Below the pacer, participants were shown a real-time visual trace of their heart rate which appeared green during periods of high resonance and amber during low resonance (see Fig. 1a). Participants had the option of enabling or disabling both the recorded breaths and the heart rate trace (by tapping the smartphone screen). At the end of the practice participants were shown summary feedback detailing the session duration and time spent in high and low resonance.

The first session of the intervention featured additional audio-based instructions that guided participants through the session. Participants were instructed to sit comfortably, relaxing their face, neck and shoulders, and to take slow, comfortable breaths in sync with the visual pacer. After settling into a comfortable breathing rhythm, participants were instructed to pay attention to the heart rate trace and attempt to stay in the green (high resonance) zone. Participants were re-assured that achieving high resonance takes practice, that it was okay to take breaks if they began to experience feelings of frustration, and that they should focus on breathing in and out fully until they were ready to re-focus on the tracer. The instructions also featured a brief explanation of the relationship between heart rate, breathing, and the body’s ability to self-regulate.

### Patient-Reported Outcomes

The primary outcome was clinically significant improvement in depression (response rate) from pre- to post-intervention, analyzed as a binary variable for which improvement was defined as ≥ 50% reduction in PHQ-9 score combined with a post-intervention score < 10 (Kroenke et al. 2001). We chose to analyze clinically significant improvement as a binary variable as we conjectured this would be most relevant to clinicians and patients, and this approach has been widely used in clinical trials for depression (Cipriani et al. 2018; Cuijpers et al. 2010; Kessler et al. 2009; Moleiro and Beutler 2009). We also analyzed PHQ-9 scores as a continuous variable, and report average (mean and median) change in PHQ-9 scores for each group at each time-point as a secondary outcome.

#### Engagement Metrics

For participants in each group, we report: the number of complete MMs, CBT/BAT exercises, and videos viewed (including introductions to each week of the intervention and other psychoeducational videos); the total volume of MM practice (in hours); the total number of messages sent by participants to their therapist, and the total number of messages received by participants from their therapist. For participants in the enhanced intervention we also report: the total number of complete HRV-B practices, the total duration (in hours) of HRV-B practice, and the average number and duration of HRV-B practices per week of the intervention.

### Statistical Analysis

Descriptive statistics were calculated for participant demographics, engagement metrics, and attrition rate in each group. For each participant, we calculated two summary measures of intervention engagement. The first involved summing the total number of complete app-based exercises common to both groups (including MM, CBT, and BAT exercises), known as “total complete exercises”. The second involved summing the total volume of meditation for participants in the standard group, and total combined volume of meditation and HRV-B practice for participants in the enhanced group, known as “total duration of practice”. We used repeated-measures ANOVA with Huynh–Feldt adjustment to examine changes in HRV-B practice over time for participants in the enhanced group. Outcome measures were analyzed using an intention-to-treat (ITT) approach in which all participants were included, regardless of intervention engagement or attrition (though we excluded n = 2 participants who did not start the intervention and who did not complete a baseline PHQ-9 measure).

For the primary outcome, we used logistic regression to compare the binary outcome of clinically significant improvement in PHQ-9 at post-intervention (coded as 1 for improvement, 0 for no improvement) between groups (coded as 1 for enhanced, 0 for standard). We used both simple logistic regression and multivariate logistic regression controlling for the following covariates: (i) baseline PHQ-9 score, (ii) age, (iii) gender, (iv) antidepressant status (coded as 1 for yes, 0 for no), (v) total complete exercises (as defined above), (vi) total duration of practice (as defined above), (vii) the number of messages sent by participants to their therapist, and (viii) the number of messages received by participants from their therapist. Since there was a high degree of colinearity between total complete exercises and total volume of practice, we repeated the regression including either metric alone, or both together, which produced equivalent results with regards to the effect of group. Since we were interested in the relationship between program engagement and symptom improvement, we report the coefficient and p-value associated with total complete exercises and total volume of practice whilst including either one or the other in the model. We used last observation carried forward (LOCF) to impute missing post-intervention PHQ-9 scores. We report the odds ratio (OR) and adjusted OR, 95% CI, and *P*-value associated with clinically significant improvement in the enhanced group relative to the standard group. We also report any covariates that were significant predictors of clinically significant improvement at the 5% significance level. Finally, we report the number needed to treat (NNT) for the enhanced relative to the standard group with 95% CIs calculated using Eqs. 1 and 2 from Bender (2001).

For the secondary continuous PHQ-9 outcome, we used linear mixed effects modelling (LMM) via the “lme4” package in R, with “time” (coded as 0, 1, 3, 5, 7, and 9), “group” (enhanced or standard), and their interaction included as fixed effects, and a separate baseline for each participant (random-intercept model). We ran the model whilst treating time as linear or as a categorical variable, and report both results. We controlled for the same covariates as in the multivariate logistic regression above, with the addition of 2-way interaction terms between “time” and covariates (iv) to (vii). We repeated the analysis by both allowing the mixed effects model to account for missing data and by using LOCF to impute missing values, which produced equivalent results. We report the contrast estimate, 95% CI, and *P*-value for each fixed effect. The estimated marginal mean (EMM) and standard error for each time-point and each group was calculated using the “emmeans” package in R. We also report PHQ-9 scores according to a per protocol analysis in which only participants with complete scores were included.

We ran several exploratory analyses to investigate the relationship between participant demographics and attrition/engagement, and the relationship between volume of HRV-B practice and change in PHQ-9 scores. For the former we used multivariate logistic regression with attrition as the dependent variable (where 1 and 0 indicated the presence or absence of a post-intervention PHQ-9 score, respectively). We also used multiple linear regression with total complete exercises, or total duration of practice as the dependent variable. In each case the explanatory variables included baseline PHQ-9 score, age, gender, antidepressant status, and group (enhanced or standard).

For the latter, using data from the enhanced group only, we used multiple linear regression and multivariate logistic regression to test whether total HRV-B engagement (in hours) predicted PHQ-9 score change or the presence of a clinically significant reduction in PHQ-9 score, respectively, whilst controlling for all covariates listed in the primary outcome analysis. Since volume of HRV-B practice was correlated with other engagement metrics, we repeated the analysis with and without including total complete exercises and total volume of meditation as covariates, and report both results.

### Sample Size

Since the present study used existing real-world data from the *Meru Health* online clinic, an a priori power calculation was not applicable. We conducted a post-hoc power calculation for the primary outcome which suggested that we achieved 0.79 power with a total sample size of n = 96 and an adjusted OR of 3.44 at an alpha level of 0.05 (two-tailed).

### Effect Size Calculation

We computed a Cohen’s *d* effect size and 95% CI for the primary outcome by converting the odds ratio using Eqs. 15 and 17 from Sánchez-Meca et al. (2003).

## Results

### Participants and Program Adherence

Participant demographics are presented in Table 1. The majority of participants were based in Finland (90.6%) and approximately one-third were taking an antidepressant prior to enrollment. Average PHQ-9 scores at baseline were similar between groups (two-sample t-test, *P* = 0.58), and at the boundary between moderate and moderately-severe depression (mean = 15.2, SD 3.69). On average, relative to the standard group, participants in the enhanced group were marginally but significantly older (mean difference = 2.9 years; two-sample t-test, *P* = 0.03), and comprised a slightly lower proportion of males and those taking antidepressant medication. However, differences in gender (χ^2^ = 3.35, *P* = 0.07), antidepressant status (χ^2^ = 0.74, *P* = 0.39), and country (χ^2^ = 0, *P* = 1) were not significantly different between groups.

**Table 1:** Participant demographics

	All participants (n = 96)	Enhanced (n = 48)	Standard (n = 48)
Baseline PHQ-9 (mean, SD)	15.3 (3.69)	15.5 (3.68)	15.1 (3.73)
Age (mean, SD)	34.3 (6.51)	35.8 (6.34)	32.9 (6.41)
Gender			
Female (n, %)	78 (81.3)	43 (89.6)	35 (72.9)
Male (n, %)	18 (18.7)	5 (10.4)	13 (27.1)
Antidepressants			
Yes (n, %)	33 (34.4)	14 (29.2)	19 (39.6)
No (n, %)	63 (65.6)	34 (70.8)	29 (60.4)
Country			
Finland (n, %)	87 (90.6)	43 (89.6)	44 (91.7)
US (n, %)	9 (9.4)	5 (10.2)	4 (8.3)

*PHQ* Patient Health Questionnaire, *SD* standard deviation, *US* United States

The number of participants with a complete post-intervention PHQ-9 score was 40 (83.3%) in the enhanced group and 35 (72.9%) in the standard group (χ^2^ = 0.98, *P* = 0.32). Participants with higher PHQ-9 scores at baseline were less likely to have a complete post-intervention PHQ-9 score (b = − 0.16, *P* = 0.041). No other covariates were predictive of attrition.

### Intervention Engagement

Summary engagement metrics for each group are shown in Table 2. Across the 8-week intervention, participants completed an average of 34.5 meditations totaling 7.82 h, viewed 17.7 videos (including introductions to each week of the intervention and other psychoeducational videos), and completed 29.6 CBT/BAT exercises. Participants also sent approximately 20 messages (across 12 separate days) to their therapist and received approximately 46 messages (across 25 separate days) from their therapist. Relative to the standard group, participants in the enhanced group completed more CBT/BAT exercises (t(94) = 2.59, *P* = 0.01) and more psychoeducational videos (t(94) = 2.80, *P* = 0.01), but spent less time meditating (mean difference = 2.33 h, t(94) = − 1.61, *P* = 0.11), though the latter did not reach statistical significance (likely due to substantial individual variation in hours spent meditating; range = 0—29.2 h). In addition, females were likely to complete a higher total number of intervention practices (including CBT/BAT, MM, and psychoeducational videos [b = 30.8, *P* = 0.013]), and engage in a higher total duration of meditation practice (b = 3.93, *P* = 0.017), than males.

**Table 2:** Participant engagement

	All participant (n = 96)	Enhanced (n = 48)	Standard (n = 48)
No. of complete meditations			
Mean (SEM)	34.5 (2.71)	34.3 (3.66)	34.7 (4.02)
Min–max	0–143	1–133	0–143
Total meditation hours			
Mean (SEM)	7.82 (0.62)	6.84 (0.74)	8.81 (0.97)
Min–max	0–29.2	0–26.3	0.28–29.2
No. of complete CBT/BAT exercises			
Mean (SEM)	29.6 (1.77)	34.0 (2.43)	25.1 (2.44)
Min–max	0–78	0–78	0–56
No. of complete psychoeducation videos			
Mean	17.7 (0.61)	19.4 (0.72)	16.1 (0.93)
Min–max	3–27	4–27	3–25
No. of messages sent			
to therapist			
Mean (SEM)	19.9 (1.87)	21.7 (2.71)	18.1 (2.57)
Min–max	0–99	0–99	0–71
No. of messages received from therapist			
Mean (SEM)	46.8 (2.47)	47.7 (3.69)	46.0 (3.32)
Min–max	0–132	0–132	5–101
Total HRV-B hours			
Mean (SEM)	n/a	3.86 (0.47)	n/a
Min–max	n/a	0–13	n/a

*SEM* standard error of the mean

### HRV-B Engagement

Participants in the enhanced group completed a total average of 3.86 h of HRV-B practice across 25.8 sessions. Both the number of sessions completed (*F*_(7,329)_ = 31.4, *P* < 0.001) and the average number of minutes of HRV-B practice (*F*_(7,329)_ = 8.34, *P* < 0.001) decreased across the intervention (see Table 3). However, the mean duration of each HRV-B session completed increased from approximately 6 min to 11 min, consistent with the structure of the intervention.

**Table 3:** HRV-B engagement

	Week							
	1	2	3	4	5	6	7	8
Minutes of practice, mean (SEM)	34.6 (2.85)	30.2 (3.39)	39.2 (4.13)	31.6 (4.86)	31.9 (4.33)	20.2 (3.64)	20.6 (3.93)	23.6 (5.56)
No. of complete sessions, mean (SEM)	5.67 (0.40)	4.27 (0.40)	3.98 (0.39)	3.10 (0.45)	2.79 (0.35)	1.81 (0.33)	2.02 (0.35)	2.10 (0.48)
Duration per session in minutes, mean (SEM)	5.92 (0.17)	6.78 (0.33)	9.64 (0.40)	9.93 (0.57)	11.1 (0.40)	11.4 (0.60)	10.1 (0.56)	11.2 (0.60)

*SEM* standard error of the mean

### Patient-Reported Outcomes

In an ITT analysis, simple logistic regression revealed that participants in the enhanced group were more likely to report a clinically significant improvement in PHQ-9 score post-intervention than participants in the standard group (56.3% versus 29.2%; OR 3.12, 95% CI [1.34–7.26], *P* = 0.013). This effect remained robust when using multivariate logistic regression to adjust for participant demographics and differences in engagement between groups (adjusted OR = 3.44, 95% CI [1.28–9.26], *P* = 0.015). The difference in proportions reaching clinically significant improvement in PHQ-9 symptoms yielded a number needed to treat (NNT) of 4 (95% CI [2 to 12]) and a Cohen’s d effect size of 0.63 (95% CI [0.16 to 1.09]). In addition, participants not taking antidepressants (b = − 1.18, *P* = 0.031), and those who completed more intervention exercises (“total complete exercises”, b = 0.02, *P* = 0.024) were more likely to report a clinically significant improvement in PHQ-9. A similar pattern emerged if we included total duration of practice instead of total complete exercises in the model (see Methods; b = 0.07, *P* = 0.055).

Linear mixed effects modelling of PHQ-9 scores revealed a significant main effect of time (b = − 0.81, 95% CI [− 1.16 to − 0.46], *P* < 0.001) and a group x time interaction (b = − 0.22, 95% CI [− 0.44 to − 0.01], *P* = 0.043), suggesting that on average participants in the enhanced group experienced larger reductions in PHQ-9 scores over time than the standard group. When using the more conservative approach of treating ‘time’ as categorical (and thus not assuming a linear trend), PHQ-9 was only significantly different between groups at week 5 (mean difference = 2.20, 95% CI 0.41 to 3.99, *P* = 0.018) of the intervention (see Table 4; also see Fig. S1 for pre-post change in PHQ-9 across individual participants).

**Table 4:** Secondary patient-reported outcome means (SEM)

	Time-point					
	Baseline	w1	w3	w5	w7	Post
Standard group						
ITT mean	15.41 (0.63)	15.04 (0.64)	12.31 (0.70)	11.66 (0.71)	10.41 (0.71)	8.81 (0.70)
ITT median	15	14	11	12	10.5	10
PP mean	15.08 (0.68)	14.47 (0.81)	12.00 (0.77)	11.00 (0.78)	10.00 (0.96)	8.37 (0.99)
PP median	15	14	11	10	8.5	8
Enhanced group						
ITT mean	16.57 (0.71)	15.09 (0.71)15.09 (0.71)	12.24 (0.72)	10.61* (0.72)	10.09 (0.74)	9.16 (0.74)
ITT median	15	13	11	9	9	7
PP mean	15.50 (0.62)	13.98 (0.63)	11.22 (0.79)	9.23 (0.67)	8.80 (0.68)(0.68)	7.85 (0.76)
PP median	15	13	11	8	9	6

*PP* per protocol, *ITT* intent to treat, *SEM* standard error of the mean, *w* week

### HRV-B Engagement And Patient-Reported Outcomes

Within the enhanced group, participants who completed a higher total volume of HRV-B practice were likely to experience larger reductions in PHQ-9 symptoms (b = 0.68, *P* = 0.037). This remained marginally significant when controlling for engagement with other intervention practices, despite an increase in multicollinearity (b = 0.64, *P* = 0.053). However, HRV-B engagement did not predict the likelihood of clinically significant improvement when analyzing PHQ-9 change as a binary outcome (b = 0.2, *P* = 0.16).

## Discussion

The present study evaluated the feasibility and impact of incorporating HRV-B training into a smartphone-based, remotely-delivered, psychological intervention (*Meru Health*) for depression. We sought to determine if the enhanced intervention (including HRV-B) was associated with a higher probability of clinically significant improvement in depression relative to delivering the intervention without HRV-B. Using historical outcome data from the standard intervention as a comparison group, the present results suggest that adding HRV-B to the intervention is both feasible and associated with improved treatment outcomes.

HRV-B has seen a recent increase in popularity and has shown promise in effectuating improved outcomes across a range of disorders (Blase et al. 2016; Gevirtz 2013). However, to our knowledge this is the first demonstration of HRV-B being delivered remotely as an adjunct to a smartphone-based intervention for depression. A major benefit of app-based interventions is the ability to track participant engagement objectively. Here, participants with elevated symptoms of depression were able to complete a total of almost 4 h of HRV-B practice across 26 sessions, suggesting that the addition of HRV-B is both feasible and acceptable.

Although participants were able to increase the duration of each HRV-B session over time, the frequency (and thus overall volume of HRV-B practice) decreased across intervention weeks. This is consistent with previous reports that participants meditate less frequency over the course of the intervention (Goldin et al. 2019) and suggests that decreases in engagement are not specific to individual components of the intervention. Such global decreases in engagement may stem from fatigue, boredom, or a reduction in motivation. One of the authors (RG) has observed better adherence to daily HRV-B practice when in-session feedback included elements of gamification that rewarded participants for achieving optimal resonance. Future research should focus on understanding obstacles to HRV-B engagement, modifying the intervention to account for such obstacles, and developing methods to support sustained HRV-B practice.

Participants in the enhanced HRV-B group completed fewer meditations than participants in the standard group, which is consistent with the adapted structure of the enhanced intervention. However, HRV-B participants also engaged with significantly more CBT/BAT exercises, and more weekly introduction videos than those in the standard group. This higher pattern of engagement was unexpected and deserves additional investigation. One possibility is that the increase in intervention components in the enhanced HRV-B group helped to maintain participants’ attention and reduce fatigue. However, it’s also plausible that the increase in engagement was due to factors outside of the research group’s control, and further work is needed to establish whether higher engagement in the enhanced group remains robust in larger samples and under more controlled conditions.

The finding that participants in the enhanced group were more likely to experience clinically significant improvements in PHQ-9 compared to the standard group is consistent with other preliminary evidence that HRV-B is an effective means to reduce symptoms of depression (Karavidas et al. 2007; Patron et al. 2012; Rene et al. 2011; Siepmann et al. 2008; Steffen et al. 2017; Zucker et al. 2009). In particular, one recent study reported decreases in depression following a 6-week HRV-B intervention for participants with major depression (Lin et al. 2019), whilst another reported that combining psychotherapy with HRV-B was more effective in reducing symptoms of depression over a 6-week period than psychotherapy alone (Caldwell and Steffen 2018). The present study suggests that combining HRV-B with other evidence-based psychological treatments for depression may improve treatment outcomes, even when treatment is delivered remotely. This is particularly promising given that the standard *Meru Health* intervention (without HRV-B) has already been associated with clinically significant reductions in symptoms of depression that persist for at least one-year post-intervention (Economides et al. 2019; Goldin et al. 2019). Although the present study was not a randomized trial, and we thus cannot be sure that the difference between groups was causally related to HRV-B, the fact that participants who engaged with a higher volume of HRV-B practice were also likely to experience larger reductions in depression symptoms lends further support to the idea that HRV-B may be an effective component of depression treatment. These findings underscore the need for additional studies of HRV-B in randomized controlled settings to better understand its efficacy and to explore the mechanisms responsible for its impact on symptoms of depression.

Whilst the enhanced intervention was associated with higher odds of clinically significant improvement than the standard group, differences in the average (mean) reduction in PHQ-9 scores between groups was less clear. Since a proportion of participants reported extreme score changes, and the sample size in the present study was modest, mean changes in score might mask underlying differences between intervention groups. In particular, one participant in the enhanced group reported an extreme worsening of symptoms, whilst three participants in the standard group reported an extreme improvement in symptoms (see Fig. S1). Indeed, when using median values the difference in pre-post score-change between groups was more evident. However, we caution the reader not to overinterpret the present results. Although our study suggests that the enhanced intervention was more consistently associated with clinically significant improvements in depression than the standard intervention, further work is needed to understand whether there is an overall difference in efficacy between groups at both post-intervention and into the future. Presumably, HRV-B is a skill that, once mastered, can become routine. Given the high likelihood of relapse of depression, additional studies are needed to investigate the impact of HRV-B on such long-term outcomes.

Exploratory analyses revealed a number of results worth considering. First, participants who reported taking antidepressants at the start of the intervention were *less* likely to experience clinically significant improvements (regardless of intervention group), even after accounting for baseline differences in symptom severity. This is surprising given evidence that psychotherapy combined with pharmacotherapy is somewhat more effective than psychotherapy alone at relieving symptoms in the short-term (Cuijpers et al. 2015). One possibility is that participants taking antidepressants may have been more likely to be experiencing recurrent or persistent symptoms of depression (rather than having a first-depressive episode), and thus less susceptible to treatment. However, given a modest sample size and limited information on the type and timeframe of antidepressant usage, further work is needed in order to expand upon this finding. Moreover, although gender was not predictive of symptom reduction associated with the intervention, female engagement was higher across the intervention than male engagement. Whilst males are less likely than females to seek help for depression (Galdas et al. 2005; Kessler et al. 2005), there is little evidence to suggest that males enrolled in online interventions will engage less. Given that just 19% of the participants in the present study were male, further research is needed to understand any gender-based differences in the delivery and/or outcomes associated with the Meru Health intervention.

## Limitations

Our promising findings should be interpreted in light of several limitations. We used a non-equivalent groups design comparing an enhanced version of the Meru Heart intervention to historical outcome data from the standard intervention. Thus, participants were not randomly allocated, the researchers were not blinded to group, and we did not include a conventional control group. Despite attempting to match participants across important attributes and adjust for potential confounders in our regression models, we cannot exclude the possibility that our results were driven by differences between groups at baseline, and not by the addition of HRV-B to the enhanced intervention.

Similarly, engagement with intervention practices common to both groups (such as CBT and BAT components) was higher in the enhanced group than the standard group, which may have driven the difference in treatment outcomes between groups. However, our results remained robust when controlling for both participant demographics and intervention engagement across all analyses. Moreover, participants who engaged with HRV-B the most experienced the largest improvement in symptoms, suggesting that HRV-B may have played an active role in treatment outcomes.

Further, although engagement with HRV-B was generally high, we did not assess the extent to which participants were able to modulate their heart rate, or breath at resonance frequency during HRV-B. In addition, we did not examine whether participants in the enhanced intervention experienced increases in HRV across the intervention, or whether any such increase was related to symptom reduction. This is important because current treatments for depression do not resolve lowered HRV, even after successful treatment of depressive symptoms (Kemp et al. 2010). Future studies designed to investigate these questions are essential if we are to fully understand the mechanisms and efficacy of the enhanced intervention in treating symptoms of depression.

Lastly, the present study included self-selected participants who may have been more motivated to engage with the intervention than average. Also, the majority of participants were female, whilst male participants were found to engage with the intervention less frequently. Future studies should use more varied recruitment strategies to ensure that the present results generalize to larger and more diverse study samples.

## Conclusions

In conclusion, our findings suggest that adding HRV-B to an app-based, smartphone-delivered remote digital intervention for depression is both feasible and effective in reducing depressive symptoms. Given the public health burden of depression and potential to address access issues plaguing many who suffer from depression, the remote application of HRV-B for depression treatment is highly promising.

## Electronic supplementary material

Below is the link to the electronic supplementary material.

Electronic supplementary material 1 (DOCX 242 kb)
