# Supplementary material for: Feasibility and Efficacy of the Addition of Heart Rate Variability Biofeedback to a Remote Digital Health Intervention for Depression
Source: Appl Psychophysiol Biofeedback. 2020 Apr 3;45(2):75–86. doi: 10.1007/s10484-020-09458-z (PMC7250954; doi:10.1007/s10484-020-09458-z)
Supplement: Supplementary file 1 — Electronic supplementary material 1 (DOCX 242 kb) [file 10484_2020_9458_MOESM1_ESM.docx]

Supplementary Material


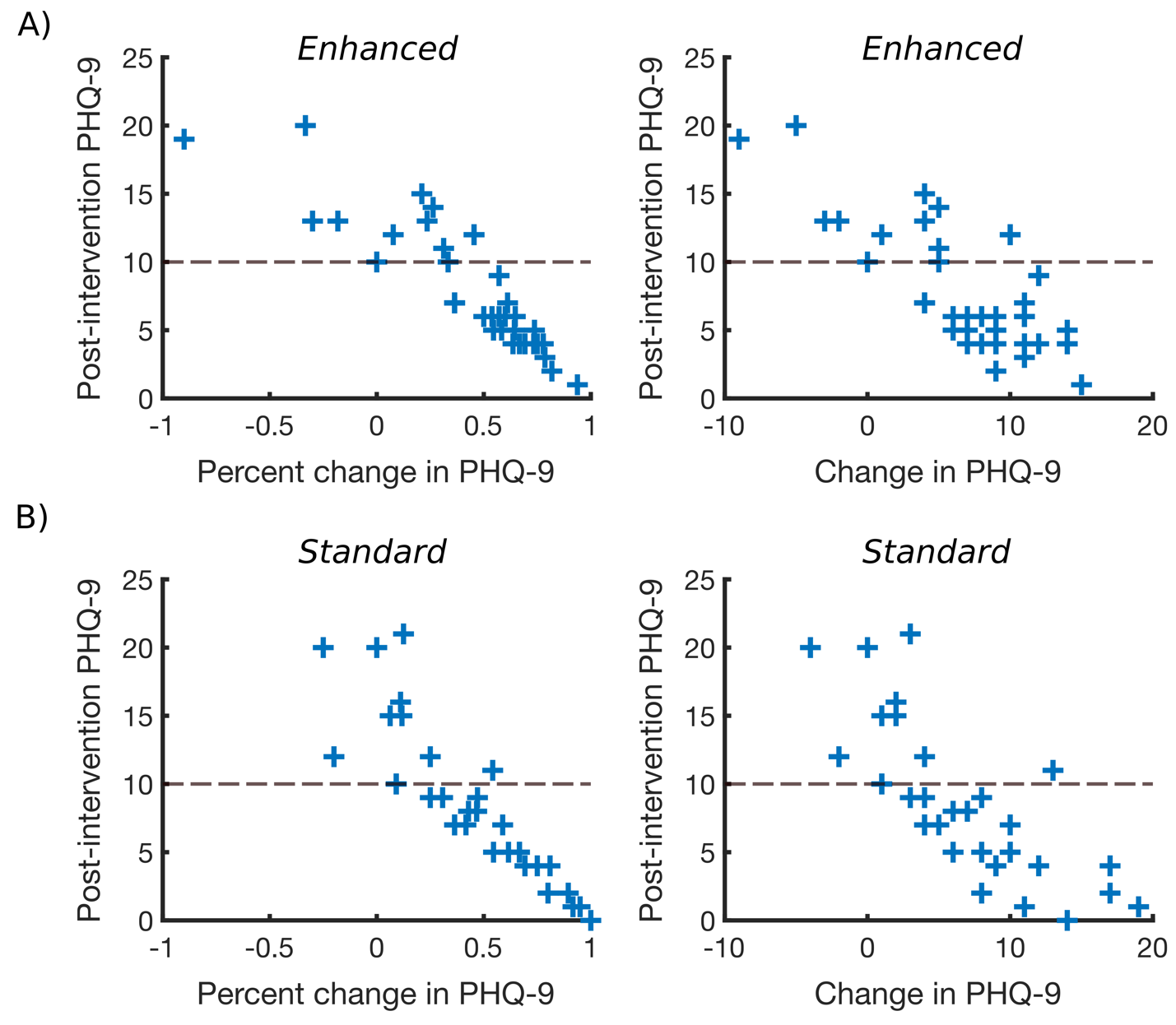


**Figure S1. Individual pre-post symptom change**. Change (right side) and percent change (left side) in PHQ-9 scores for A) the enhanced group, and B) the standard group, in participants with complete data only (per protocol analysis). Each symbol represents a single participant. The horizontal dotted line represents the post-intervention PHQ-9 threshold for clinically significant improvement (PHQ < 10).
